# Emotion Modulation through Music after Sadness Induction—The Iso Principle in a Controlled Experimental Study

**DOI:** 10.3390/ijerph182312486

**Published:** 2021-11-26

**Authors:** Katrin Starcke, Johanna Mayr, Richard von Georgi

**Affiliations:** 1 SRH Berlin School of Popular Arts (SOPA), Media Psychology, Potsdamer Straße 188, 10783 Berlin, Germany; jojo.mayr@googlemail.com (J.M.); richard.von-georgi@srh.de (R.v.G.); 2 Berlin Institute of Biomusicology and Empirical Research (BIBER), 10783 Berlin, Germany

**Keywords:** music, emotion modulation, iso principle, personality, affect

## Abstract

Music therapy intervention manuals suggest that individuals who suffer from affective disorders benefit from listening to music according to the iso principle. The iso principle comprises listening to music that matches the current mood of patients at first, and then to gradually shift to music that represents a desired mood. Within the current study, we investigate whether the sequence of music with different emotional valence can modulate the emotional state. All participants were healthy adults who underwent a sadness induction via a movie clip. They were subsequently divided into four experimental groups. Each was asked to listen to two pieces of music according to a specific sequence: sad-sad; sad-happy; happy-happy; happy-sad. Participants were prompt to rate their current emotional state at different stages of the experiment: prior to and after the movie clip, as well as after each of the two pieces of music. The frame used for the assessment was the Positive and Negative Affect Schedule and the Self-Assessment Manikin. The results indicate that the movie clip induced sadness. The group of participants who listened to the sad music first and the happy music afterwards ultimately reported a higher positive affect, a higher emotional valence, and a lower negative affect compared with the other groups. However, not all the between-group differences reached significance. We conclude that the sequence of music with different emotional valence affects the current emotional state. The results are generally in line with the iso principle. Directions for future research are presented.

## 1. Introduction

There has been a debate whether music elicits emotions in the listeners or whether listeners simply recognize the emotion expressed by the piece of music [1]. There are numerous studies which emphasize the power of music to elicit emotions in the listener. An early study [1] assessed the emotional and physiological reactions towards music and discovered that different music elicits different emotional states and different physiological arousal. Subsequent studies have also demonstrated the emotion-inducing effects of music [2]. Music is used to induce emotions in various psychological disciplines [3]. Mechanisms of how exactly music induces emotions have been summarized recently [4], namely through brain stem reflexes (quick and basic auditory sensations mediating arousal, attention, and judgment of consonance and dissonance), evaluative conditioning (repeated pairing of pieces of music with other positive or negative stimuli), emotional contagion (perceiving the emotional expression of the music and mimicking this expression), visual imagery (generating visual images while listening to the music), episodic memory (evoking a memory of a specific event through a piece of music), and expectancy (the music violates, delays, or confirms the expectancy on how the music continues). These mechanisms are not working separately, and specific pieces of music induce emotions by the interplay of the mechanisms proposed. Some mechanisms, for example evaluative conditioning and episodic memory, explain why individuals can be differently affected by the same music. Nevertheless, some acoustical properties could be identified which are perceived similar in the listeners [5]. Happiness is associated with fast tempo, major mode, simple and consonant harmony, medium-high sound level, high pitch, ascending pitch, smooth and fluent rhythm, or bright timbre. Sadness is associated with slow tempo, minor mode, low sound level, low pitch, descending pitch, flat or falling intonation, dull timbre, legato, or less energetic execution. To what extent a perceived emotion really induces the corresponding emotion depends on the listeners appraisal and the current reason for listening to music, but also on biological-based reactions towards music. An example of an intense emotional experience during music listening which strongly relies on subcortical structures is the experience of chills [6]. Chills are intense sensations experienced during music listening which are mainly experienced as rewarding by the listeners [7]. Chills can include shivers down the spine, lump in the throat, goosebumps, trembling, and sexual arousal [8]. During chill experiences, a typical physiological and neural pattern could be observed. On a peripheral physiological level, skin conductance responses occur, heart rate increases, respiration increases, and pupillary dilation increases, which all represent a heightened arousal [9,10,11,12]. On the brain level, chills are associated with increased activation within the mesolimbic reward system, which emphasizes their rewarding nature [13,14].

However, not only happy music can induce chills, but sad music can also induce chills and can be experienced as rewarding. Although humans generally avoid sadness in everyday life, sad music (and art in general) are explicitly strived for and can be experienced as pleasurable. Thus, sadness in an aesthetic context can be pleasurable. A recent review summarizes potential mechanisms through which sad music elicits pleasure and reward [15]. The authors suggested that music-induced sadness is pleasurable when it is perceived as non-threatening, when it is aesthetically pleasing, and when it elicits psychological advantageous mechanisms such as mood regulation. Thus, sad music is considered capable of correcting a negative emotional state by the aesthetical pleasure, and by empathetic feelings due to music listening which may be caused by the reflection of past events. The topic of mood regulation through music has also been addressed in a recent set of studies [16,17,18]. The authors suggest mechanisms of mood regulation through music which are applicable for both happy and sad music. The mechanisms include happy mood maintenance, psyching up and energizing, revival and relaxation, strong sensation, diversion and distraction, discharge and disclosure, solace, and mental work. Mental work describes facing, contemplating, reappraising, and working through disturbing emotional experiences. In an alternative model of mood regulation by music [19], the basic dimensions relaxation with music, cognitive problem solving, reduction of negative activation, positive stimulation, and arousal modulation are connected with health and subjective well-being [20,21], psychiatric illness [22], and music therapy-related changes [23]. Existing studies on the use of music in everyday life show that music allows to go deeper into the emotional experience and resolve the negative emotion towards building a better emotional state. This is in line with the empathetic feelings and reflection of past events, which was proposed by Sachs et al. [15] as an underlying mechanism of why sad music also has positive effects. These mechanisms also play a role in music therapeutical interventions.

Music listening has been utilized for the treatment of various clinical conditions [24]. Apart from active music therapeutic interventions, listening to music has also been shown to be useful in the treatment of affective disorders, anxiety disorders, and various stress-related health problems. A recent meta-analysis implies that listening to music leads to short-term beneficial effects in patients with depression [25]. An important mechanism that underlies the therapeutic effects of music is the modulation of emotions through music. An early theoretical approach suggested that patients should listen to pieces of music according to the iso principle [26]. It means that patients listen to music which is aligned with their current emotional state (i.e., a negative one), and from there on, shift to music expressing a desired state (i.e., a positive one). The sequence of music should help the patients to integrate the internal and external experiences, and shift to a more positive perspective and experience. The iso principle has been included in modern music therapy intervention manuals [27]. It has also been transferred to other creative therapies such as dance therapy [28,29]. In the 1950s, the iso principle was mainly associated with the tempo of the music [30]. It was proposed that the tempo and mood of the music fits to the “mental tempo” of the subject. From the 1970s on, the emphasis of the iso principle clearly shifted to the emotional state of the subject [31]. Today, the iso principle is one possible explanation of how people use music in everyday life to regulate their emotions and feelings. Nevertheless, there is currently no empirically based theory that explains the emotional shift. Therefore, the iso principle is defined as the fit between person (emotion, affect, and arousal) and music variables (e.g., tempo and mode). A recent review summarized the history of the iso principle in music therapy [32]. In addition, it demonstrates a therapeutic progress after music listening according to the iso principle in a patient who suffered from depression. Although the underlying theoretical concept of the iso principle is accepted and applied in music therapy, controlled experimental studies are scarce. To the best of our knowledge, only one experimental study exists [31] which showed the first evidence for the iso principle. However, the study had several shortcomings, such as the lack of control conditions in which music was listened to in a different order than in the iso principle. Furthermore, dependent variables were used to assess only broad categories of emotional experiences and only male subjects participated in the study. The current study fills this research gap and investigates with a controlled experimental design whether the sequence of music with happy and sad valence modulates current mood. In our experimental study, we only included healthy participants who underwent a sadness induction first.

The reactions towards music should also depend on participants’ personality. The results of a previous study [33] found relationships between music-induced emotions and the Big Five personality factors [34]. Specifically, they found medium correlations between pleasure-enjoyment and neuroticism, anxiety-fear and conscientiousness, and a negative correlation between pleasure-enjoyment and openness. The authors emphasize that other factors such as the environment also influence the currently experienced emotions.

We aimed to investigate the effects of listening to music with different emotional valence in different sequences. We did not investigate patients with affective diseases such as depression but included healthy participants who underwent a sadness induction. After the sadness induction, they listened to two pieces of music in a specific sequence. Participants were divided into four experimental groups, each listening to one sequence of music: sad-sad; sad-happy; happy-happy; happy-sad. According to the iso principle, we expected the group who listened to a sad piece first, and a happy piece afterwards, to have the most positive emotional state afterwards. This group is called the iso-group below. For exploratory reasons, we analyzed effects of trait affect, personality, and gender.

## 2. Materials and Methods

Overall, 107 participants took part in the study, of which 68 were female, 38 male, and 1 non-binary. Participants’ age ranged from 18 to 76 (M = 30.58, SD = 12.34). Because of the sadness induction during the study, exclusion criteria were affective disorders, anxiety disorders, and other psychiatric disorders which may worsen due to the study. In addition, participants who were younger than 18 years old were also excluded from the study. All participants clicked a button that they agreed with the use of the data for scientific purposes. Then, they clicked a button that they meet the inclusion criteria and that they understood that they may quit the study at any point if they wish to do so. The study started if these two buttons were clicked. After finishing the experiment, participants were fully debriefed and thanked for participation. The study was approved by the local ethics committee. For optimal sample size calculation, we used the study by Shatin [31] as a reference. Although the study has some shortcomings and the statistics are not completely reported, the results suggest a large effect size. This information was incorporated into the program G*Power 3.1.9.2. (Faul, Erdfelder, Lang & Buchner, 2007, Heinrich Heine University, Düsseldorf, Germany) [35]. We selected a fixed effects ANOVA without repeated measures as the basis for the calculation because the comparison between the four groups’ emotional state at the end of the experiment was the main analysis of interest. With a large effect size, an alpha-error probability of 0.05, and four independent groups, the optimal sample size was calculated to be 112. We were finally able to receive 107 complete datasets. One participant reported in the “comments” field that he could not hear the movie clip properly, and two participants reported to be younger than 18 years old. These three datasets were excluded from the analysis. In addition, 22 participants did not finish the study and their data were not analyzed either.

During the COVID-19 crisis, the study was conducted online via the tool LabVanced (Scicovery GmbH, Paderborn, Germany) [36]. The study was password protected to ensure that only the desired recipients took part in the study. The study started off with the measurement of preferred genres, general affective state, and personality as baseline measures. After that, the sadness induction took place, followed by the two pieces of music. The participants were randomly assigned to one of four experimental groups, who differed in the order of music they were exposed to: sad-sad; sad-happy (iso-group); happy-happy; happy-sad. During the experiment, participants were monitored regarding their emotional state. They rated their current emotional state prior to the sadness induction, after the sadness induction, after the first piece of music, and after the second piece of music.

To measure genre preferences, we used a German translation of the Short Test of Music Preferences (STOMP) [37]. In the first part of this questionnaire, participants had to choose their favorite amongst 16 genres (classical, blues, heavy metal, soundtracks/theme songs, modern classical/avantgarde, schlager, country/western, rap/hip-hop, jazz, alternative, pop, soul/funk, Latin/reggae, rock, religious, and techno/dance). The music preferences can be subdivided into four main categories: category one “reflexive and complex” includes genres such as classical, jazz, blues, and folk; category two “intense and rebellious” includes genres such as rock, heavy metal, and alternative; category three “upbeat and conventional” includes genres such as country, pop, and religious music; and category four “energetic and rhythmic” includes genres such as rap/hip-hop, soul/funk, and electronic/dance. Participants’ preferences according to these four categories were reported in the results section of the current study to demonstrate that the four experimental groups did not differ in regard to their preference.

To measure trait positive and negative affect, we used the German version of the Positive and Negative Affect Schedule (PANAS) [38]. It consists of ten adjectives that represent positive affect and ten adjectives that represent negative affect. Each adjective is answered on a five-point Likert scale ranging from 1 (not at all) to 5 (extremely).

To measure participants’ personality, we used the Short Eysenck Personality Profiler with NEO-PI-R Openness (SEPPO) [39], which is a modified version of the Eysencks Personality Profiler (EPPD) [40] plus the dimension openness to experiences from the NEO Five Factor Inventory [34]. Thus, the questionnaire assesses the four personality dimensions extraversion, neuroticism, psychoticism, and openness to experiences. It includes 21 items which can be answered on a five-point Likert scale from 1 (does not apply) to 5 (applies).

In addition, the participants’ use of music in everyday life has been assessed with the Inventory for the assessment of Activation and Arousal modulation through Music (IAAM) [19,41]. However, results were not used for analyses in the current study.

For the induction of sadness, a movie clip from the movie “The Champ” (1979) was used. It lasted approximately three minutes and included a scene from the final part of the movie. The protagonist “Champ” has completed a boxing mach. He has won the match but is severely injured. He lies on a couch and dies at the end of the scene. His young son thinks he is sleeping and tries to wake him up. The scene has successfully been used for sadness induction in previous studies [42].

Four pieces of music were used in the current study; two of them were happy and two of them were sad. The two happy pieces were “Blue Danube” (Johann Strauss, 1867), and the Romance from “A little Night Music” (Wolfgang Amadeus Mozart, 1787). The two sad pieces were “Kol Nidrei” (Max Bruch, 1880), and “Suite in A-Minor” second movement (Christian Sinding, 1889). The pieces were mainly chosen on the basis of a previous study which reported the respective valence ratings and accompanying brain activations [43]. In contrast to this previous study, we changed the movement of “A little Night Music”: while in the previous study the “Allegro” and “Rondo Allegro” were used, we used the “Romance” movement. This was done in order to have a somewhat similar tempo between all four pieces of music. Each of the pieces was played for about 90 s.

To measure emotional state during the experiment, participants rated their current emotional valence with the Self-Assessment Manikin (SAM) [44,45]. The rating scale consists of a nine-point scale, and each point is associated with a manikin representing an emotional valence from negative to positive. The Self- Assessment Manikin comprise two further nine-point scales which assess current arousal and current perceived dominance. These two scales were also applied, but not included in the analyses of the current study. Furthermore, participants rated their current emotional state in more detail with the state version of the Positive and Negative Affect Schedule (PANAS) [38]. As described above, it consists of ten adjectives that represent positive affect and ten adjectives that represent negative affect. Each adjective is answered on a five-point Likert scale ranging from 1 (not at all) to 5 (extremely).

Potential differences between the four experimental groups concerning gender and music preferences were analyzed with *X*^2^ tests. Potential group differences concerning age and trait variables were analyzed with ANOVAs. Between-within interactions among the four groups x the points of measurement concerning emotional state were analyzed with repeated measures ANOVAs (4 × 2 for the sadness induction; 4 × 3 for the effects of music exposure). Greenhouse Geisser corrections were applied when appropriate. Differences between groups concerning the current emotional state at the final point of measurement were assessed with univariate ANOVAs and *LSD* post-hoc tests. Partial eta squared was used as an effect size for all ANOVAs. For the analyses of relationships between trait affect and personality with the emotional state, we performed Pearson correlations.

## 3. Results

### 3.1. Demographic Variables and Trait Measures

Results of the demographic variables, music preferences, trait affect, and personality of the four experimental groups are shown in Table 1. The results indicate that the groups did not differ concerning age (*F* = 1.41, *df* = 3103, *p* = 0.25), gender (*X*^2^ = 5.31, *df* = 6, *p* = 0.51), music preferences (*X*^2^ = 10.63, *df* = 9, *p* = 0.30), trait positive affect (*F* = 1.09, *df* = 3, 103, *p* = 0.36), trait negative affect (*F* = 0.10, *df* = 3, 103, *p* = 0.96), neuroticism (*F* = 0.97, *df* = 3, 103, *p* = 0.41), extraversion (*F* = 1.23, *df* = 3, 103, *p* = 0.30), psychoticism (*F* = 1.29, *df* = 3, 103, *p* = 0.28), or openness (*F* = 1.69, *df* = 3, 103, *p* = 0.17). The results demonstrate successful randomization of participants across the experimental groups.

### 3.2. Effects of Sadness Induction

**Figure 1:**
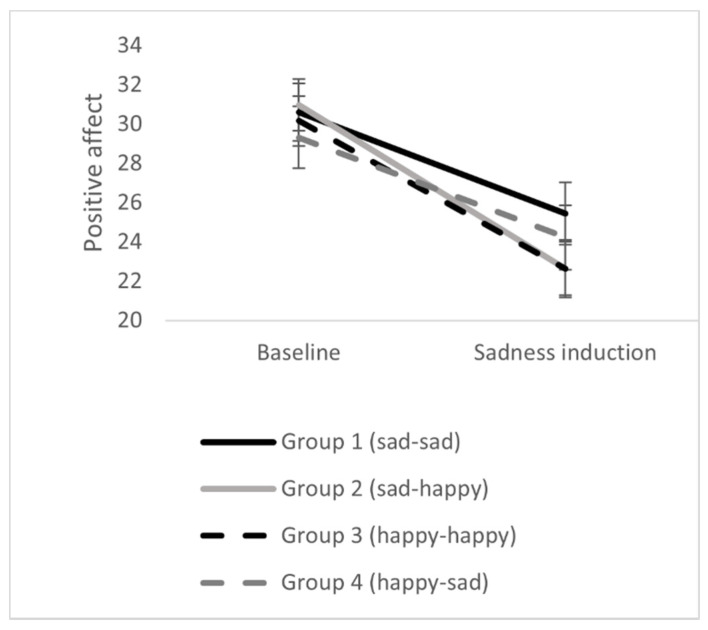
Effects of sadness induction on positive affect in the four experimental groups. Error bars represent standard errors.

**Figure 2:**
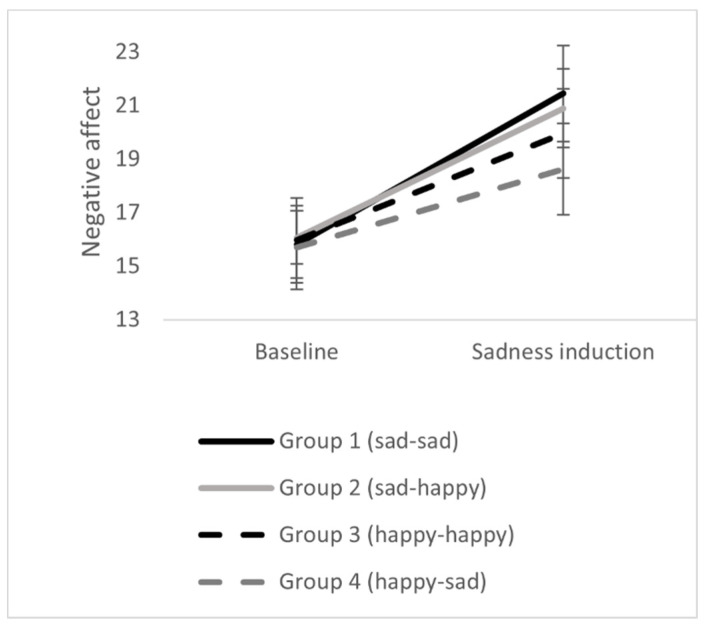
Effects of sadness induction on negative affect in the four experimental groups. Error bars represent standard errors.

**Figure 3:**
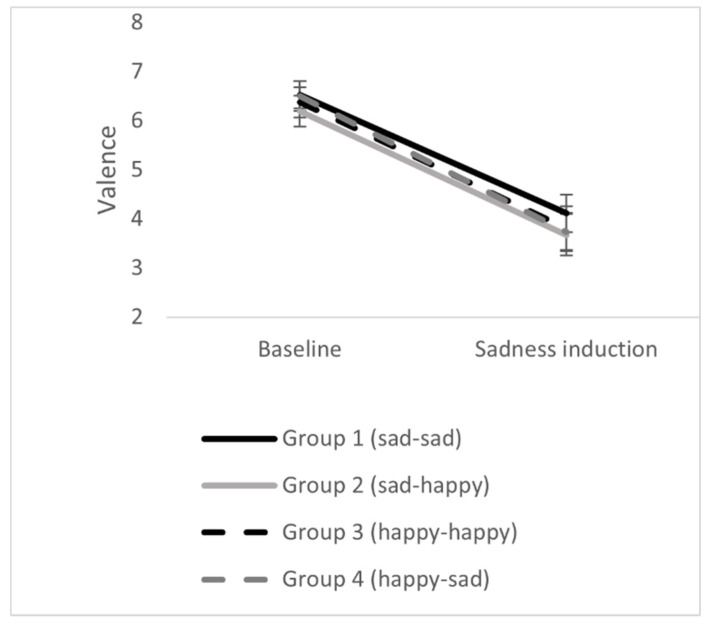
Effects of sadness induction on emotional valence in the four experimental groups. Error bars represent standard errors.

The sadness induction via the movie clip was successful. In all four groups positive affect decreased, emotional valence decreased, and negative affect increased significantly. There were no differences between the four experimental groups. Thus, the sadness induction worked in all experimental groups. The results can be seen in Table 2 and Figure 1, Figure 2 and Figure 3.

### 3.3. Effects of Music Listening

**Figure 4:**
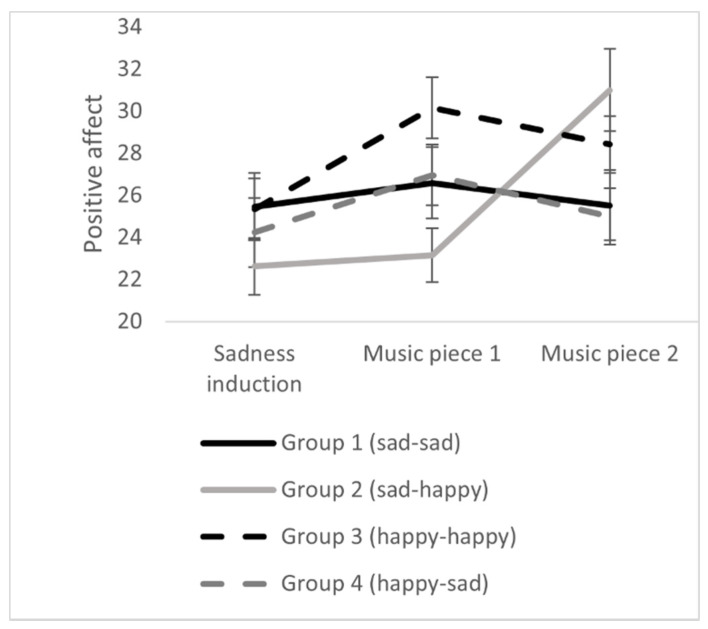
Effects of music listening on positive affect in the four experimental groups. Error bars represent standard errors.

**Figure 5:**
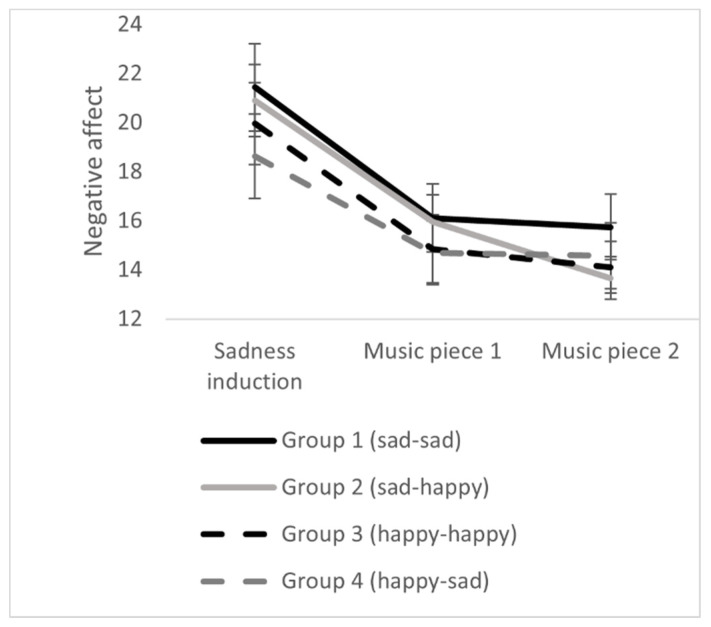
Effects of music listening on negative affect in the four experimental groups. Error bars represent standard errors.

**Figure 6:**
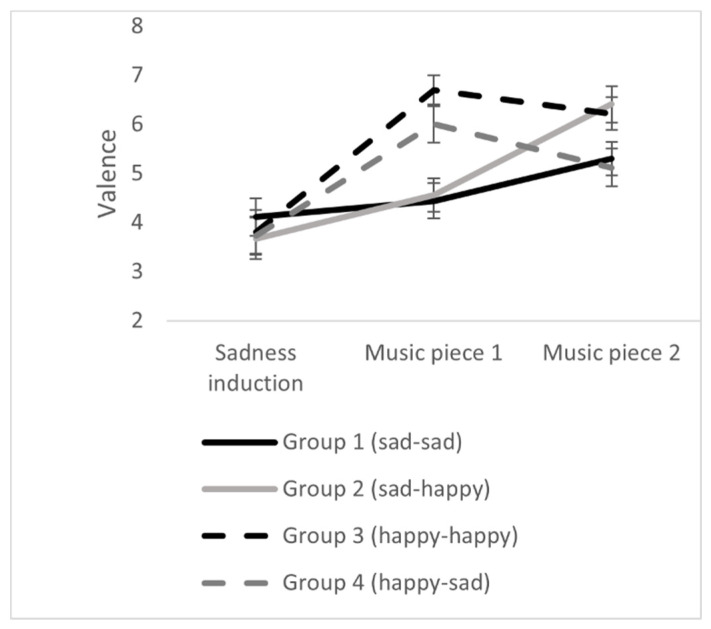
Effects of music listening on emotional valence in the four experimental groups. Error bars represent standard errors.

Results demonstrate significant changes concerning positive affect, negative affect, and emotional valence during the exposure to the two pieces of music. Concerning positive affect and valence, there were significant interactions between group x point of measurement. The results can be seen in Table 3 and Figure 4, Figure 5 and Figure 6. The results indicated that the iso-group (sad-happy) finally had the highest positive affect, the highest emotional valence, and the lowest negative affect. Thus, on a descriptive level, results are in line with the iso principle. This effect will now be analyzed in detail.

### 3.4. The Iso Principle Effect in Detail

We now compare the emotional state after listening to the two pieces of music in detail. It was of particular interest to investigate whether the iso-group (sad-happy) differed from the other three groups. Univariate ANOVAs indicate that the four groups differ significantly concerning positive affect (*F* = 2.97, *df* = 3, 103, *p* < 0.05, *η*^2^ = 0.08) and emotional valence (*F* = 3.33, *df* = *3*, 103, *p* < 0.05, *η*^2^ = 0.09) after music listening. *LSD* post-hoc tests show that the iso-group had higher positive affect than group 1 (sad-sad; mean difference = 5.48, *SE* = 2.27, *p* < 0.05, 95% confidence interval = 0.97–9.99) and group 4 (happy-sad; mean difference = 6.00, *SE* = 2.30, *p* = 0.01, 95% confidence interval = 1.45–10.55). The iso-group also showed a more positive emotional valence compared to group 1 (sad-sad; mean difference = 1.11, *SE* = 0.50, *p* < 0.05, 95% confidence interval = 0.12–2.10) and group 4 (happy-sad: mean difference = 1.29, *SE* = 0.51, *p* = 0.01, 95% confidence interval = 0.29–2.29). The comparison between the iso-group (sad-happy) and group 3 (happy-happy) points in the same direction with higher values in positive affect and emotional valence in the iso-group on a descriptive level, but the difference fails to reach significance.

We finally re-ran the ANOVAs in which we compared the four experimental groups concerning their final emotional state (positive affect, negative affect, and valence) separately for males and females. The results indicated that the iso principle effect was not observed in males, but only in females. Within the female subgroup, results indicate that the iso-group had a higher positive affect than group 1 (sad-sad; mean difference = 6.05, *SE* = 2.70, *p* < 0.05, 95% confidence interval = 0.65–11.44), group 4 (happy-sad; mean difference = 5.89, *SE* = 2.99, *p* = 0.05, 95% confidence interval = −0.08–11.86), and a trend towards higher positive affect than group 3 (happy-happy; mean difference = 4.89, *SE* = 2.74, *p* = 0.08, 95% confidence interval = −0.58–10.36), and a higher valence than group 1 (sad-sad; mean difference = 1.57, *SE*= 0.60, *p* = 0.01, 95% confidence interval = 0.38–2.77) and group 4 (happy-sad; mean difference = 1.97, *SE* = 0.66, *p* < 0.005, 95% confidence interval = 0.64–3.29).

### 3.5. Relationship between Emotional State after the Manipulations, Personality, and Trait Affect

We calculated the relationships between the three emotional state variables (positive affect, negative affect, and emotional valence) after music listening with trait positive and negative affect, and the personality traits neuroticism, extraversion, psychoticism, and openness. This was done for the four experimental groups separately. In all experimental groups, state positive affect was related to trait positive affect, and state negative affect was related to trait negative affect (all *p*s < 0.05). Within the iso-group (sad-happy), there was a significant negative relationship between state negative affect and extraversion (*r* = −0.42, *p* < 0.05). Within group 4 (happy-sad), there was a significant relationship between state positive affect and extraversion (*r* = 0.50, *p* = 0.01), state negative affect and neuroticism (*r* = 0.56, *p* < 0.005), and state positive affect and openness (*r* = 0.48, *p* = 0.01). Within group 1 (sad-sad) and group 3 (happy-happy), no significant relationships between emotional states and personality were observed.

## 4. Discussion

Results indicate that the movie clip induced sadness and that listening to music afterwards changed the emotional states. In line with our hypothesis, participants of the iso-group had the highest positive affect, the highest emotional valence, and the lowest negative affect. Significant differences were observed for positive affect and emotional valence between the iso-group and the groups sad-sad and happy-sad. The most interesting comparison, whether the iso-group differed from the group who listened to happy pieces only, pointed in the same direction, but results did not reach significance. In the subgroup of female participants, a trend towards a significant difference between the iso-group and the happy-only group was observed. In the subgroup of male participants, the effect was not observed.

Results indicate that listening to music for mood regulation according to the iso principle might have some advantages over listening to happy music only or to other sequences of happy and sad music. The iso principle suggests that listening to music which matches the current mood first picks individuals up where they are; listening to music with a different (and desired) valence afterwards helps them to shift to a desired perspective and experience. In case of patients who suffer from depression and who are in a sad mood, this means listening to sad music first, and listening to happy music afterwards. In the current study, only healthy participants who underwent a sadness induction were included. Thus, the negative affective state which is chronically present in patients who suffer from depression was partially simulated by the acute induction of sadness. Current results are principally in line with the ideas of Altshuler [26], the single case study by Heiderscheit and Madson [32], and the study by Shatin [31]. The study by Shatin was conducted with healthy young males who were not exposed to sadness. The study did not include control conditions (i.e., music in other sequences than according to the iso principle) and can, therefore, be regarded as a first attempt to investigate the iso principle experimentally. The study by Heiderscheit and Madson includes a single female patient who suffered from depression, anxiety, and overeating disorder. She profited from listening to individually selected music according to the iso principle. This study has a high ecological validity but includes only a single patient.

The current study was experimental in nature with a design that includes different sequences of happy and sad music. This experimentally sophisticated design bears the disadvantage of a lower ecological validity. Thus, no patients were included, participants only listened to two pieces of music, and participants could not select the music themselves. Despite these limitations, the results emphasize that the iso principle might work. The results indicate that this might be particularly the case in females. In addition, interesting relationships between state affect after music listening and personality were detected. In the two experimental groups that were exposed to happy and sad music (the iso-group and the happy-sad group), high positive and low negative affect were positively related to high extraversion, high openness, and low neuroticism. In the two experimental groups that were exposed to either happy or sad music only, no relationships between the final affective state and personality were observed. In these groups, states induced by the music might have exceeded individual differences.

## 5. Conclusions

We conclude that the results of the current study principally support the iso principle. All measures of current emotional state (positive affect, negative affect, and valence) were in line with the iso principle, i.e., the participants of the iso-group had the highest positive affect, the lowest negative affect, and the highest valence after listening to the two pieces of music. However, not all between-group comparisons reached significance, and future research should address several points. Future research should particularly concentrate on comparing an iso-group with a happy-only group. In the current study, a trend towards a difference has been detected, but only in females. It would be essential to investigate the iso principle in a larger sample with an equal number of male and female participants to determine potential gender differences in detail. With the experience of the current study, the number of participants should be increased. In addition, it would be of interest to include more than two pieces of music to have a more gradual shift from sad to happy music in the iso-group. It would also be of interest to include music from different genres and not only classical pieces, or to include music that is selected individually by the participants. Doing so, one might give advice for optimizing the usage of the iso principle, for example regarding which genres are particularly suited or whether self-selected or therapist-selected music is most advantageous. Apart from assessing current states via a questionnaire, it would also be an option to include psychophysiological measures during music listening, such as heart rate or electrodermal activity. This would be particularly of interest when examining not only the valence dimension of emotional state, but also the arousal dimension. For therapeutic usage, it would also be essential to find out how long the effects of application of the iso principle last. Thus, the emotional state should be monitored for a period after the music exposure. Finally, the iso principle should be tested in patient groups including male and female patients with comprehensive documentation.

## Figures and Tables

**Table 1:** Demographic variables, music preferences, and personality.

	Group 1 Sad-Sad	Group 2 Sad-Happy	Group 3 Happy-Happy	Group 4 Happy-Sad
N	27	27	27	26
Number of females	19	18	18	13
Age	28.19 (9.46)	28.15 (9.68)	33.19 (16.51)	32.88 (12.10)
Reflexive and complex	2	3	5	4
Intense and rebellious	4	6	5	2
Upbeat and conventional	7	3	5	11
Energetic and rhythmic	14	15	12	9
Trait positive affect	36.22 (5.17)	33.52 (5.56)	35.30 (5.50)	33.88 (8.32)
Trait negative affect	18.85 (5.48)	19.74 (7.68)	19.03 (6.04)	18.92 (7.65)
Neuroticism	15.30 (3.48)	15.81 (3.74)	14.56 (3.17)	14.35 (3.82)
Extraversion	20.04 (2.33)	19.30 (3.00)	19.07 (2.96)	18.58 (2.97)
Psychoticism	PP17.15 (2.27)	15.78 (3.18)	16.07 (2.70)	16.15 (2.68)
Openness	22.33 (3.66)	22.30 (3.35)	23.07 (4.04)	20.81 (3.93)

**Table 2:** Results of the sadness induction.

	*F*	*df*	*MSE*	*p*	*η_p_^2^*
Positive affect point of measurement	86.98	1, 103	21.00	<0.001	0.46
Positive affect group	0.24	3, 103	94.05	0.87	0.01
Positive affect group × point of measurement	1.78	3, 103	21.00	0.16	0.05
Negative affect point of measurement	41.05	1, 103	24.56	<0.001	0.29
Negative affect group	0.24	3, 103	99.62	0.87	0.01
Negative affect group × point of measurement	0.72	3, 103	24.56	0.54	0.02
Valence point of measurement	144.56	1, 103	2.43	<0.001	0.58
Valence group	0.31	3, 103	4.44	0.82	0.01
Valence group × point of measurement	0.12	3, 103	2.43	0.95	<0.01

**Table 3:** Results of the emotional state after music listening.

	*F*	*df*	*MSE*	*p*	*η_p_^2^*
Positive affect point of measurement	11.00	1.74, 179.46	28.53	<0.001	0.10
Positive affect group	0.81	3, 103	140.43	0.49	0.02
Positive affect group × point of measurement	7.25	5.23, 179.46	28.53	<0.001	0.17
Negative affect point of measurement	79.26	1.43, 147.27	17.80	<0.001	0.44
Negative affect group	0.38	3, 103	129.65	0.77	0.01
Negative affect group × point of measurement	1.06	4.29, 147.27	17.80	0.38	0.03
Valence point of measurement	58.74	1.82, 187.45	2.13	<0.001	0.36
Valence group	1.93	3, 103	7.00	0.13	0.05
Valence group × point of measurement	8.21	5.46, 187.45	2.13	<0.001	0.19

## Data Availability

Data are accessible via Mendeley Data: Starcke, Katrin (2021), “Iso principle”, Mendeley Data, V1, doi: 10.17632/3bt9tz83pm.1 https://data.mendeley.com//datasets/bhbkzx46fn/1 (accessed on 23 October 2021).

## References

[1] Krumhansl C.L. (1998). An exploratory study of musical emotions. Can. J. Exp. Psychol..

[2] Altenmüller E., Kopiez R. Starke Emotionen und Gänsehaut Beim Musikhören: Evolutionäre und musikpsychologische Aspekte. Proceedings of the 16. Multidisziplinäres Kolloquium der GEERS-STIFTUNG.

[3] Västfjäll D. (2002). Emotion induction through music: A review of the musical mood induction procedure. Music. Sci..

[4] Juslin P.N., Västfjäll D. (2008). Emotional responses to music: The need to consider underlying mechanisms. Behav. Brain Sci..

[5] Juslin P.N., Laukka P. (2004). Expression, perception, and induction of musical emotions: A review and a questionnaire study of everyday listening. J. New Music Res..

[6] Zatorre R.J., Salimpoor V.N. (2013). From perception to pleasure: Music and its neural substrates. Proc. Natl. Acad. Sci. USA.

[7] Goldstein A. (1980). Thrills in response to music and other stimuli. Physiol. Psychol..

[8] Kunkel M., Pramstaller M.C., Grant P., von Georgi R. (2008). Ein Konstruktpsychologischer Ansatz zur Messung des Chill-Erlebens. Samples. www.aspm-samples.de/Samples7/kunkeletal.pdf.

[9] Laeng B., Eidet L.M., Sulutvedt U., Panksepp J. (2016). Music chills: The eye pupil as a mirror to music’s soul. Cosciousness Cogn..

[10] Rickard N.S. (2004). Intense emotional responses to music: A test of the physiological arousal hypothesis. Psychol. Music.

[11] Salimpoor V.N., Benevoy M., Longo G., Cooperstock J.R., Zatorre R. (2009). The rewarding aspects of music listening are related to degree of emotional arousal. PLoS ONE.

[12] Starcke K., von Georgi R., Tiihonen T.M., Laczika K.-F., Reuter C. (2019). Don’t drink and chill: Effects of alcohol on subjective and physiological reactions during music listening and their relationships with personality and listening habits. Int. J. Psychophysiol..

[13] Blood A.J., Zatorre R.J. (2001). Intensely pleasurable responses to music correlate with activity in brain regions implicated in reward and emotion. Proc. Natl. Acad. Sci. USA.

[14] Salimpoor V.N., Benevoy M., Larcher K., Dagher A., Zatorre R. (2011). Anatomically distinct dopamine release during anticipation and experience of peak emotion to music. Nat. Neurosci..

[15] Sachs M.E., Damasio A., Habibi A. (2015). The pleasure of sad music: A systematic review. Front. Hum. Neurosci..

[16] Saarikallio S.H. (2010). Music as emotional self-regulation throughout adulthood. Psychol. Music.

[17] Saarikallio S.H., Erkkilä J. (2007). The role of music in adolescents’ mood regulation. Psychol. Music.

[18] Saarikallio S.H. (2008). Music in mood regulation: Initial scale development. Music. Sci..

[19] Von Georgi R., Grant P., von Georgi S., Gebhardt S. (2006). Personality, Emotion and the Use of Music in Everyday Life: Measurement, Theory and Neurophysiological Aspects of a Missing Link.

[20] Herr J., von Georgi R., Kreuz G., Bernatzky G. (2021). A critical contribution to the disease-promoting effect of youth-specific music genres. Music and Medicine.

[21] Von Georgi R., Cimbal K., von Georgi S. (2009). Aktivations-und Arousal-Modulation Mittels Musik im Alltag und Deren Beziehungen zu Musikalischen Präferenzen, Persönlichkeit und Gesundheit. Musikpsychologie—Musikalisches Gedächtnis und Musikalisches Lernen.

[22] Gebhardt S., Kunkel M., von Georgi R. (2014). Emotion modulation in psychiatric patients through music. Music Percept..

[23] Gebhardt S., Dammann I., Loescher K., Vedder H., von Georgi R. (2018). The effects of music therapy on the interaction of the self and emotions—An interim analysis. Complement. Ther. Med..

[24] Pauwels E.K.J., Volterrani D., Mariani G., Kostkiewics M. (2014). Mozart, music and medicine. Med. Princ. Prax..

[25] Aalbers S., Fusar-Poli L., Freeman R.E., Spreen M., Ket J.C.F., Vink A.C., Maratos A., Crawford M., Chen X.-J., Gold C. (2017). Music therapy for depression. Cochrane Database Syst. Rev..

[26] Altshuler I.M. (1944). Four years’ experience with music as a therapeutic agent at Eloise Hospital. Am. J. Psychiatry.

[27] Wigram T., Pedersen I., Bonde L. (2002). A Comprehensive Guide to Music Therapy: Theory, Clinical Practice, Research, and Training.

[28] Trautmann-Voigt S., Voigt B. (2020). Grammatik der Körpersprache: Ein Integratives Lehr-und Arbeitsbuch zum Embodiement.

[29] Trautmann-Voigt S., Starcke K. (2020). Personal communication.

[30] Ruppenthal W.W. (1954). Experimental determinants of perception. Some considerations for music therapy. Music Ther..

[31] Shatin L. (1970). Alteration of mood via music: A study of the vectoring effect. J. Psychol..

[32] Heiderscheit A., Madson A. (2015). Use of the iso principle as a central method in mood management: A music psychotherapy clinical case study. Music Ther. Perspect..

[33] Juslin P.N., Liljeström S., Västfjäll D., Barradas G., Silva A. (2008). An experience sampling study of emotional reactions to music: Listener, music, and situation. Emotion.

[34] Costa P.T.J., McCrae R.R. (1992). Revised NEO Personality Inventory (NEO PI-R) and NEO Five-factor Inventory (NEO-FFI): Professional Manual.

[35] Faul F., Erdfelder E., Lang A.-G., Buchner A. (2007). G*Power 3: A flexible statistical power analysis program for the social, behavioral and biomedical sciences. Behav. Res. Methods.

[36] Labvanced (2020). Scicovery GmbH, Paderborn, Germany. www.labvanced.com.

[37] Rentfrow P.J., Gosling S.D. (2003). The do re mi’s of everyday life: The structure and personality correlates of music preferences. J. Pers. Soc. Psychol..

[38] Breyer B., Bluemke M. (2016). German Version of the Positive and Negative Affect Schedule PANAS.

[39] Von Georgi R., Herr J. Short Eysenck personality profiler with NEO-PI-R openness.

[40] Eysenck H.J., Jackson C.J., Bultheller S., Wilson G.D. (1998). Eysenck Personality Profiler: EPP-D Manual.

[41] Von Georgi R. (2013). Use of Music in Every-Day Life: Theory and Validation Studies with the IAAM.

[42] Gross J.J., Levenson R.W. (1995). Emotion elicitation using films. Cogn. Emot..

[43] Mitterschiffthaler M.T., Fu C.H.Y., Dalton J.A., Andrew C.M., Williams S.C.R. (2007). A functional MRI study of happy and sad affective states induced by classical music. Hum. Brain Mapp..

[44] Bradley M.M., Lang P.J. (1994). Measuring emotion: The self-assessment manikin and the semantic differential. J. Behav. Ther. Exp. Psychiatry.

[45] Lang P.J., Bradley M.M., Cuthbert B.N. (1997). International Affective Picture System (IAPS): Technical Manual and Affective Ratings. NIMH Cent. Study Emot. Atten..

